# DNA double-strand breaks in human induced pluripotent stem cell reprogramming and long-term in vitro culturing

**DOI:** 10.1186/s13287-017-0522-5

**Published:** 2017-03-21

**Authors:** Pavel Simara, Lenka Tesarova, Daniela Rehakova, Pavel Matula, Stanislav Stejskal, Ales Hampl, Irena Koutna

**Affiliations:** 1 Centre for Biomedical Image Analysis, Faculty of Informatics, Masaryk University, Kamenice 5, 625 00 Brno, Czech Republic; 2 Department of Histology and Embryology, Faculty of Medicine, Masaryk University, Kamenice 3, 625 00 Brno, Czech Republic

**Keywords:** Human induced pluripotent stem cells, DNA double-strand breaks, γH2AX, 53BP1, Long-term in vitro culture, DNA repair

## Abstract

**Background:**

Human induced pluripotent stem cells (hiPSCs) play roles in both disease modelling and regenerative medicine. It is critical that the genomic integrity of the cells remains intact and that the DNA repair systems are fully functional. In this article, we focused on the detection of DNA double-strand breaks (DSBs) by phosphorylated histone H2AX (known as γH2AX) and p53-binding protein 1 (53BP1) in three distinct lines of hiPSCs, their source cells, and one line of human embryonic stem cells (hESCs).

**Methods:**

We measured spontaneously occurring DSBs throughout the process of fibroblast reprogramming and during long-term in vitro culturing. To assess the variations in the functionality of the DNA repair system among the samples, the number of DSBs induced by γ-irradiation and the decrease over time was analysed. The foci number was detected by fluorescence microscopy separately for the G1 and S/G2 cell cycle phases.

**Results:**

We demonstrated that fibroblasts contained a low number of non-replication-related DSBs, while this number increased after reprogramming into hiPSCs and then decreased again after long-term in vitro passaging. The artificial induction of DSBs revealed that the repair mechanisms function well in the source cells and hiPSCs at low passages, but fail to recognize a substantial proportion of DSBs at high passages.

**Conclusions:**

Our observations suggest that cellular reprogramming increases the DSB number but that the repair mechanism functions well. However, after prolonged in vitro culturing of hiPSCs, the repair capacity decreases.

**Electronic supplementary material:**

The online version of this article (doi:10.1186/s13287-017-0522-5) contains supplementary material, which is available to authorized users.

## Background

Human induced pluripotent stem cells (hiPSCs) hold great promise for clinical applications because of their potential to differentiate into all three embryonic germ layers [1–3]. To use hiPSCs in cell therapy or disease modelling [4], it is fundamental that they possess an intact genome. Much research has been performed in the field of genome maintenance in mouse and human embryonic stem cells (hESCs). However, less is known about the causes of genomic aberrations and the functionality of repair mechanisms in hiPSCs [5]. In general, the genomic instabilities in hiPSCs may be introduced: 1) by pre-existing mutations in source cells; 2) during reprogramming; and 3) during in vitro expansion of the hiPSCs. It has been reported that at least 50% of the single-nucleotide variations in hiPSCs pre-existed in the source cells [6]. The process of reprogramming itself represents a serious risk of mutation acquisition. Primarily, deletions of tumour suppressor genes were observed during reprogramming [7]. Using episomal vectors may lower the risk of reprogramming-associated genome changes [8]. The culturing of pluripotent stem cells (PSCs) in vitro is probably the main cause of the accumulation of genomic instabilities, resulting from the adaptation to culture conditions and clonal selection during passaging. The data reported by Taapken et al. [9] support this idea, indicating that the types and frequency of karyotypic abnormalities are similar between hESCs and hiPSCs. In contrast, the results of Laurent et al. [7] revealed slight differences in the distribution of subchromosomal variations between hESCs and hiPSCs. Interestingly, in their study [7], prolonged in vitro culturing was associated with oncogene duplication.

One of the key techniques for monitoring DNA integrity is the detection of DNA double-strand breaks (DSBs). DSBs are a severe type of DNA damage that may cause irreversible changes in the genomic content of the cell. They are induced by internal factors such as the by-products of cell metabolism or replication stress, or by external factors such as exposure to irradiation or chemical agents [10]. A damaged cell may arrest the cell cycle until the lesions are repaired. If the DNA damage is not successfully repaired, apoptosis is commonly induced to prevent the propagation of chromosomal aberrations. The repair of DSBs is executed either by fast non-homologous end-joining (NHEJ) or more precise homologous recombination (HR). Both mechanisms contribute to DSB repair in a cell cycle-specific manner. NHEJ occurs at all phases of the cell cycle but is primarily responsible for DSB repair in the G1 stage. HR occurs predominantly in the late S and G2 phases [11]. Published data suggest that pluripotent cells exert stronger genomic protection and can repair DNA lesions more efficiently than differentiated somatic cells [5, 12–14]. However, a strong DNA protective mechanism may cause the pluripotent cells to be more prone to apoptosis.

Various DNA damage-response proteins have been used as markers of DSBs, including phosphorylated histone H2AX (known as γH2AX) and p53-binding protein 1 (53BP1) [15]. The generation of γH2AX foci at the site of DNA lesions precedes the formation of 53BP1 foci [16–18]. Several studies have confirmed that 53BP1 functions exclusively in NHEJ and that it inhibits the 5′ end resection needed for HR [19–21]. In contrast, γH2AX influences both NHEJ and HR [10]. The foci formation of γH2AX is dependent on the cell cycle phase [22–24]. S/G2 phase cells exhibit more γH2AX foci than do cells in G1 phase because of replication-related DSBs. Cell-cycle dependency has not been observed for 53BP1 [25].

In the present study, we compared the genomic integrity of fibroblasts and pluripotent stem cells. We used fluorescence microscopy to visualize the DSBs recognized by γH2AX and 53BP1 in three hiPSC lines and one hESC line at low or high passage numbers and in one line of source cell fibroblasts. Each hiPSC line is unique and represents a different reprogramming approach, as described in the Methods. We also aimed to detect differences in the ability to recognize DSBs artificially induced by γ-irradiation and their decrease over time. The measurements were conducted with respect to cell cycle stage, and the data were analysed separately for the G1 and G2/S phases. Thus, we aimed to elucidate genomic stability during hiPSC generation and in vitro culturing.

## Methods

### hiPSC generation and cell culture

Human dermal fibroblasts (hDFs; kindly provided by the National Tissue Centre Inc., Brno, Czech Republic) and CD34^+^ haematopoietic progenitors (blood sample kindly provided by the Department of Internal Medicine, Haematology and Oncology, Masaryk University, and University Hospital Brno, Czech Republic) were used as source cells for the generation of hiPSCs as described in Šimara et al., 2014 [26, 27]. For this study, we used the hiPSC line CBIA-3–CD34^+^ haematopoietic progenitors reprogrammed by the Sendai virus (CytoTuneTM-iPS Reprogramming Kit; Thermo Fisher Scientific, Waltham, MA, USA), hiPSC line CBIA-5–fibroblasts reprogrammed by the Sendai virus, and hiPSC line CBIA-7–fibroblasts reprogrammed by episomal vectors (Epi5™ Episomal iPSC Reprogramming Kit; Thermo Fisher Scientific). The CCTL-14 hESC line [28] was a kind gift from the Department of Histology and Embryology (Faculty of Medicine, Masaryk University, Brno, Czech Republic). All three hiPSC lines and the hESC line were maintained in the form of colonies on irradiated mouse embryonic fibroblast feeder cells (MEFs; 2.5 × 10^5^ cells per 3.5-cm dish) in DMEM/F12 (1:1) supplemented with 20% knock-out serum replacement, 2 mM l-glutamine, 100 μM non-essential amino acids, 1% penicillin/streptomycin, 0.1 mM 2-mercaptoethanol, and 10 ng/ml basic fibroblast growth factor (bFGF) (all from Thermo Fisher Scientific). The medium was changed daily. Markers of pluripotency (Oct-3/4, Sox2, Nanog, and SSEA4) were detected as described previously [26], and all three hiPSC lines were positive for all of these markers (Additional file 1: Figure S1). A teratoma formation assay confirmed that the hiPSCs could differentiate into all three germ layers (Additional file 2: Figure S2).

The following cell passage numbers (p) were used (low and high): CBIA-3 at p27 and p76, CBIA-5 at p19 and p65, CBIA-7 at p25 and p67, and CCTL-14 at p30 and p302. hDFs were used at p6. No CD34^+^ blood progenitors used to generate CBIA-3 were available for DSB analysis.

### γ-Irradiation

Prior to irradiation, the hiPSCs and hESCs were feeder depleted by culturing on a Geltrex® matrix for 3 days. Essential 8™ medium (Thermo Fisher Scientific) was changed daily. The cells were then irradiated by ionizing radiation (IR; 0.5 Gy/min; ^137^Cs; 1 and 4 Gy) to induce DSBs and fixed in 4% paraformaldehyde at 0.5, 2, and 6 h after irradiation.

The dose of 1 Gy was selected for the experiments based on published results [12, 29] and our DSB count measurement after 1 Gy or 4 Gy irradiation (data not shown). The peak value of the foci number was recorded 0.5 h after irradiation; therefore, this time point was selected for the study of the functionality of the repair system.

### Immunocytochemistry

Immunocytochemical staining was used to visualize the DSBs and distinguish between the cell cycle stages G1 and S/G2. Four hours before fixation, a nucleoside analogue of thymidine, 5-ethynyl-2′-deoxyuridine (EdU; Thermo Fisher Scientific), was added at a final concentration of 10 μM. The cells were fixed in 4% paraformaldehyde and permeabilized in 0.2% Triton-X (both from Sigma-Aldrich, St. Louis, MO, USA). Overnight incubation with primary antibodies against γH2AX (Biolegend, San Diego, CA, USA) and 53BP1 (Santa Cruz Biotechnology, Dallas, TX, USA) was followed by 1 h of incubation with a secondary antibody conjugated with Alexa 555 (Cell Signaling Technology, Danvers, MA, USA). The samples were stained with the Click-iT® EdU Alexa Fluor® 488 Imaging Kit (Thermo Fisher Scientific) to visualize EdU according to the manufacturer’s instructions. Finally, the nuclei were stained with Hoechst dye (BisBenzemide H33342; 1 μg/ml; Sigma-Aldrich).

### Fluorescence microscopy and image analysis

Fluorescent signals were detected using the Zeiss Axiovert 200 M system (Carl Zeiss, Oberkochen, Germany). The images were captured using a CoolSNAP HQ2 CCD camera in the wide-field mode (Photometrics, Tucson, AZ) at –30 °C. Thirty 3-μm slices were acquired in each field at a resolution of 1392 × 1040 pixels. The pixel size of the images was 124 × 124 nm. Between 500 and 1000 cells were analysed from each microscopic slide. Two slides, γH2AX and 53BP1, were prepared from each sample and each time point.

Acquiarium software, developed by our group, was used to acquire and analyse the images [30]. Acquiarium is open source software available for download at our group’s official website (http://cbia.fi.muni.cz/projects/acquiarium.html). During the analysis, individual cells in the field of view were first cropped manually. Next, the nucleus of each cell, stained with Hoechst dye, was recognized automatically using the “Find objects (hysteresis thresholding)” plugin. We used the Gaussian filter in the preprocessing phase (with sigma = 1), the threshold was calculated using the two-level Otsu method, and we defined the minimum size of an object to exclude the parts of adjacent cells. This plugin defined the area in which we counted γH2AX or 53BP1 foci. For this purpose, we employed the eMax algorithm described in [31] using the parameters sigma = 1, a spot height threshold of 80, and a maximum spot size of 800, which we set empirically. The EdU signal was quantified based on the total intensity calculated in the nucleus. The threshold for the separation of EdU-negative (G1) and EdU-positive (S/G2) cells was computed in MATLAB (Mathworks) using the Otsu method.

### Flow cytometry analysis

To assess early apoptosis, cells were stained with Annexin-V fluorescein isothiocyanate (FITC) and 7-amino-actinomycin D (7-AAD; BD Via-Probe) in Annexin-V binding buffer (Miltenyi Biotec, Bergisch Gladbach, Germany). From each sample, approximately 1 × 10^5^ cells were processed. All of the samples were measured using a BD FACS Canto II flow cytometer (Becton-Dickinson). BD FACSDiva (Becton-Dickinson) software was used for the data analysis.

### Western blotting

For each time point, approximately 1 × 10^6^ cells were lysed in RIPA buffer. The total protein concentration was assessed using a Pierce™ BCA Protein Assay Kit (Thermo Fisher Scientific). Laemmli buffer was added, and the samples were separated by SDS-PAGE. The proteins were transferred onto polyvinylidene fluoride membranes, and the membranes were blocked with 5% milk in TBS-Tween for 1 h. The membranes were then incubated with a 1:1000 dilution of PARP and GAPDH primary antibodies (both from Cell Signaling Technology) in TBS-Tween with 5% milk at 4 °C overnight. Subsequently, the membranes were incubated with the secondary antibody (1:5000 anti-rabbit HRP; Cell Signaling Technology) for 1 h at room temperature, and the blots were developed using the Clarity™ Western ECL Substrate (Bio-Rad Laboratories, Hercules, CA, USA) according to the manufacturer’s instructions.

### Statistical analysis

Comparison of two data sets was performed using Student’s *t* test. Multi-group assays were analysed by a one-way analysis of variance (ANOVA) in conjunction with Tukey’s test. A level of *P* ˂ 0.05 was considered to be statistically significant.

## Results

### Discrimination between the cell cycle phases using EdU increases the accuracy of analysing DNA lesions

**Fig. 1:**
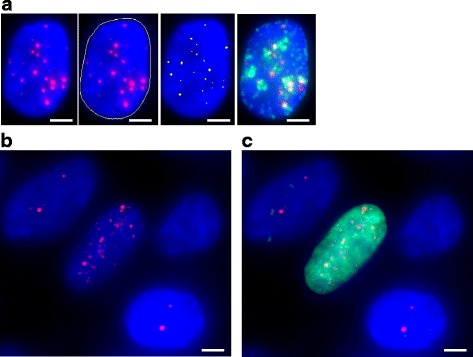
Image analysis in three dimensions using Acquiarium software. **a** Automatic detection of the cell nucleus (*blue*) marked with a *yellow border line* and counting of 21 γH2AX foci (*red*) inside the cell nucleus, visualized by *yellow dots*. The *green* regions emerge as EdU is newly incorporated during DNA synthesis. **b** A significantly higher count of γH2AX foci is seen in the nucleus of the cell in the middle of the field than in the adjacent cells. **c** The cell in the middle is strongly positive for EdU (*green*), suggesting that the cell passes through the S or G2 phase, and γH2AX foci result from replication stress. The hiPSC line CBIA-5 at low passage, non-irradiated control, γH2AX. A merge of 30 3-μm slices is shown. *Scale bar* = 5 μm. *EdU* 5-ethynyl-2′-deoxyuridine

**Fig. 2:**
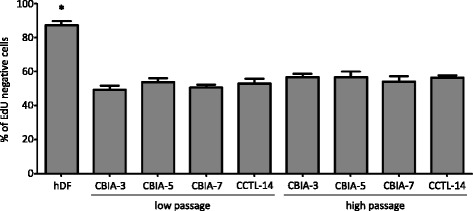
Distribution of EdU-negative cells in the samples. Comparison of fibroblasts (hDF), hiPSCs (CBIA-3, CBIA-5, and CBIA-7), and hESCs (CCTL-14) at low or high passage number. The mean value of the percentage of EdU-negative cells calculated from six histograms is shown (± SEM). A massive disproportion in the EdU-negative cell group was observed between hDF somatic cells and pluripotent stem cells. *P ˂ 0.05 by one-way ANOVA and Tukey’s multiple comparison test. *EdU* 5-ethynyl-2′-deoxyuridine, *hDF* human dermal fibroblast

The overall goal of our study was to use the numbers of γH2AX and 53BP1 foci as a measure of DNA repair in hiPSCs and in their somatic founders. However, as described above, it has been previously shown that the numbers of γH2AX foci are influenced by the cell cycle phase, with more foci being present in the S/G2 nuclei than in the G1 nuclei [22–24]. Obviously, different types of cells (somatic versus pluripotent) as well as cells in different states of culture (early versus late) most likely differ in the lengths of the individual phases of their cell cycle. Therefore, we first determined to what extent the numbers of foci are influenced by cell cycle speed and may thus distort the overall picture obtained by the foci analysis. To do so, we labelled newly synthesized DNA with EdU, visualized the accumulation of γH2AX and 53BP1 proteins on chromatin (foci), and then used an automated analysis. This approach is shown in Fig. 1a. Figure 1b and c exemplify the situation when an EdU-positive cell (nucleus) contains a larger number of γH2AX foci compared to EdU-negative cells (nuclei). Before we counted the numbers of γH2AX and 53BP1 foci, we analysed the EdU signal distribution among the cell samples and separated the EdU-negative (G1 phase) and EdU-positive (S/G2 phase) nuclei. The EdU signal strength in particular cells in each sample was then expressed as a histogram (with a calculated threshold for EdU negativity) for maximum clarity and reproducibility in separating G1 and S/G2 cells. Histograms of all analysed samples are shown in Additional file 3 (Figure S3). Our data revealed a statistically significant difference in cell cycle phase distribution between hDFs, representing a somatic cell type, and all pluripotent stem cells, irrespective of their type and passage number (Fig. 2). The high proportion (87.2%) of EdU-negative cells in the hDF sample suggests that the vast majority of these cells remain in G1 phase. By contrast, only between 49.5 and 57.0% of the pluripotent cells were EdU negative, confirming their high proliferation activity and short cell cycle.

Taken together, this series of experiments demonstrates the robustness of the approach that we have developed to visually discriminate between G1 and S/G2 cells in situ. Our data show that, using this technique, we can identify changes in cell cycle progression. In the context of cell cycle-associated differences in numbers of γH2AX and 53BP1 foci, this approach is extremely useful and was employed for all the following analyses in this study. The Acquiarium software also represents an extremely valuable tool for complex and automated microscope image analysis.

### Reprogramming is accompanied by increased numbers of γH2AX and 53BP1 foci, but this trend is reversed with prolonged in vitro culturing

**Fig. 3:**
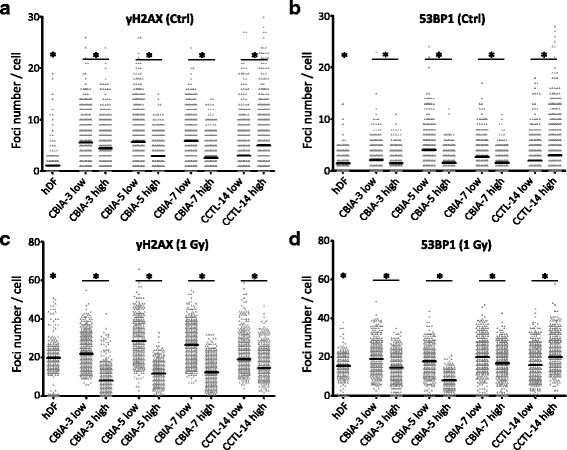
DSBs recognized by γH2AX (**a**,**c**) and p53-binding protein 1 (*53BP1*) (**b**,**d**) in non-irradiated control cells (*Ctrl*) (**a**,**b**) or 0.5 h after γ-irradiation with 1 Gy (**c**,**d**). Comparison of fibroblasts (human dermal fibroblast; *hDF*), hiPSCs (CBIA-3, CBIA-5, and CBIA-7) and hESCs (CCTL-14) at low or high passage number. The number of foci in each cell is visualized as a dot, and the mean value is shown as a black line for each sample. The results are shown in the EdU-negative population. *P ˂ 0.05 between hDF and iPSCs in low passage, and between low and high passages, by one-way ANOVA and Tukey’s multiple comparison test

First, we wanted to determine whether reprogramming to pluripotency influences the numbers of DSBs as revealed by the presence of γH2AX and 53BP1 foci. To do so, we counted these foci in the parent fibroblasts (hDFs) and in cells of three independent hiPSC lines (CBIA-3, CBIA-5, and CBIA-7) at an early stage after their establishment (up to passage 27; further referred to as low-passage hiPSCs). As shown in Fig. 3a and b, the numbers of both types of foci in EdU-negative hiPSCs were higher than those observed in EdU-negative hDFs. Specifically, in hDFs, the average number of foci per cell was only 1.1 for γH2AX and 1.5 for 53BP1. In hiPSCs, however, these numbers ranged from 5.6 to 5.9 for γH2AX and from 2.1 to 4.0 for 53BP1. It needs to be stressed that the CBIA-5 and CBIA-7 cells produced about the same numbers of γH2AX foci (5.69 and 5.89, respectively) despite the different reprogramming method used to generate these cells (Sendai virus versus episomal vectors). The next question was whether prolonged passaging of hiPSCs may further affect the number of DSBs. To obtain this information, we evaluated foci in hiPSCs (all three lines as above) that were cultured for a minimum of 65 passages (further referred to as high-passage cells). In these high-passage hiPSCs, the numbers of foci decreased (compared to low-passage cells), reaching levels of only 2.6 to 4.4 foci per cell for γH2AX and 1.5 to 1.6 foci per cell for 53BP1.

**Fig. 4:**
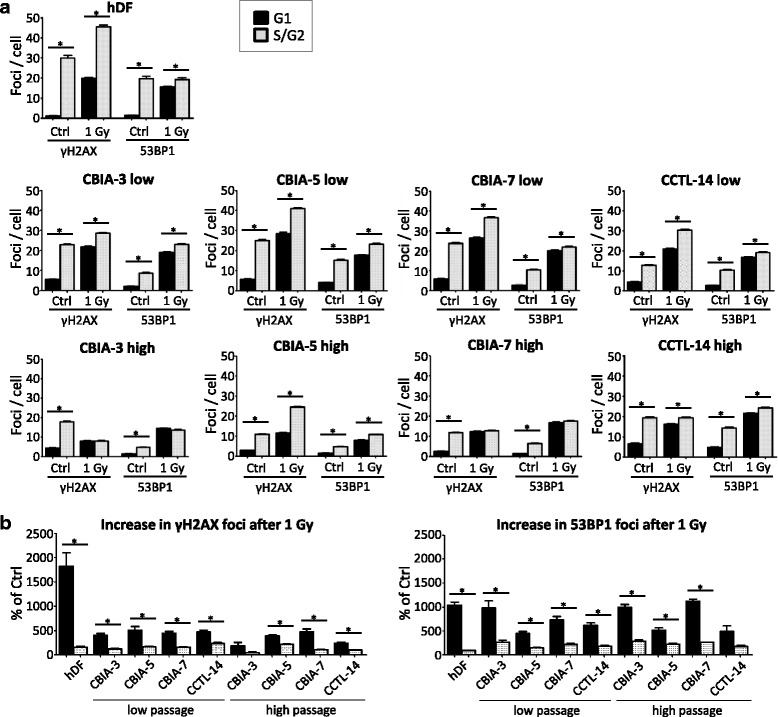
Cell cycle-dependent changes in the γH2AX and p53-binding protein 1 (*53BP1*) foci number. A comparison of the foci number between the G1 phase (Edu negative; *black column*) and S/G2 phase (EdU positive; *grey column*) was performed in one fibroblast line (human dermal fibroblast; *hDF*), three hiPSC lines (CBIA-3, CBIA-5, and CBIA-7) and one hESC line (CCTL-14) at high or low passage number. **a** The number of foci per cell is shown in non-irradiated control cells (*Ctrl*) and irradiated cells (1 Gy; 0.5 h after irradiation). **b** Percentage of increase in γH2AX and 53BP1 foci after 1 Gy treatment. The mean ± SEM is shown. *P ˂ 0.05 by *t* test

As described in the previous section, EdU-positive (S/G2) cells are characterized by many more DSBs than EdU-negative (G1 phase) cells, possibly as a result of replicative stress-associated amplification of DNA lesions during the progression of the cell cycle. Accordingly, the numbers of both γH2AX and 53BP1 foci were increased in EdU-positive cells compared to EdU-negative cells in all cell lines and passage categories (low and high) analysed in this experiment (Fig. 4). Interestingly, this S/G2-linked increase was the highest in hDFs, with the mean numbers of foci per EdU-positive cell being 29.9 for γH2AX and 19.6 for 53BP1, probably reflecting their highly effective “healthy” repair machinery. In the low-passage hiPSCs, the respective mean numbers were slightly lower than in hDFs, 23.0–25.1 for γH2AX and 8.8–15.3 for 53BP1, while in high-passage hiPSCs these numbers dropped down to 11.0–17.9 for γH2AX and 4.7–6.5 for 53BP1. It is also of note that the mean numbers of γH2AX foci were always (in all cell lines as well as passage categories) higher than those of 53BP1 foci (Fig. 4a).

Since we hypothesized that increased DSBs are due to reprogramming rather than being associated with pluripotency, we thought that hESCs would have a rather low basal level of DSBs, possibly about the same as in hDFs. To address this issue, we also analysed a reference line of hESCs (CCTL-14) that we have shown in our previous work to conform in all aspects to hESC standards [28]. Contrary to our expectation, the numbers of DSB-associated foci in new, low-passage hESCs were much closer to those in hiPSCs than in hDFs. This held true for both EdU-negative and EdU-positive cells. Specifically, in EdU-negative cells the numbers averaged 4.5 for γH2AX and 2.7 for 53BP1 foci, and in EdU-positive cells they averaged 12.8 for γH2AX and 10.3 for 53BP1 foci. Clearly, the numbers of foci in hESCs follow the same trend as in hDFs and hiPSCs, being dramatically increased in S/G2 cells compared to the cells in G1 phase. Additionally, as in hDFs and hiPSCs, the numbers of γH2AX foci in hESCs were always higher than those of 53BP1 foci. Surprisingly, however, in hESCs the numbers of γH2AX- and 53BP1-associated foci further increased with their prolonged culturing, which was in strict contrast to what we observed in hiPSCs (see above). Specifically, the numbers of foci per cell in high-passage hESCs were as follows: in the EdU-negative cells, 6.7 for γH2AX and 4.8 for 53BP1 foci; in the EdU-positive cells, 19.3 for γH2AX and 14.3 for 53BP1 foci. The complete set of foci numbers is shown in Table 1.

**Table 1:** Number of foci per cell

	Non-irradiated control cells				Irradiated cells (1Gy)			
	G1 (EdU-negative cells)		S/G2 (EdU-positive cells)		G1 (EdU-negative cells)		S/G2 (EdU-positive cells)	
	No. of foci ± SEM	No. of cells	No. of foci ± SEM	No. of cells	No. of foci ± SEM	No. of cells	No. of foci ± SEM	No. of cells
hDF								
γH2AX	1.13 ± 0.11	463	29.9 ± 1.32	60	19.89 ± 0.36	485	45.49 ± 0.88	97
53BP1	1.49 ± 0.07	486	19.64 ± 1.23	80	15.55 ± 0.22	501	19.20 ± 0.91	69
CBIA-3 low								
γH2AX	5.56 ± 0.22	464	22.98 ± 0.38	523	21.87 ± 0.34	612	28.71 ± 0.34	554
53BP1	2.13 ± 0.12	445	8.76 ± 0.35	407	19.01 ± 0.31	552	23.1 ± 0.42	496
CBIA-3 high								
γH2AX	4.42 ± 0.20	483	17.91 ± 0.41	337	8.01 ± 0.27	594	7.97 ± 0.35	480
53BP1	1.46 ± 0.07	625	4.73 ± 0.18	510	14.45 ± 0.29	551	13.66 ± 0.31	447
CBIA-5 low								
γH2AX	5.69 ± 0.23	542	25.1 ± 0.47	415	28.6 ± 0.52	437	41.00 ± 0.55	421
53BP1	4.03 ± 0.18	445	15.3 ± 0.49	425	17.84 ± 0.26	613	23.32 ± 0.30	642
CBIA-5 high								
γH2AX	2.98 ± 0.13	520	10.98 ± 0.26	508	11.57 ± 0.27	445	24.45 ± 0.39	480
53BP1	1.56 ± 0.07	608	4.71 ± 0.21	461	8.04 ± 0.17	490	10.84 ± 0.20	514
CBIA-7 low								
γH2AX	5.89 ± 0.25	382	23.77 ± 0.45	414	26.48 ± 0.44	511	36.71 ± 0.52	444
53BP1	2.73 ± 0.13	487	10.42 ± 0.31	421	20.05 ± 0.35	591	21.94 ± 0.38	560
CBIA-7 high								
γH2AX	2.56 ± 0.13	547	11.78 ± 0.25	534	12.41 ± 0.26	580	12.73 ± 0.30	452
53BP1	1.51 ± 0.07	586	6.5 ± 0.27	415	16.9 ± 0.32	556	17.62 ± 0.34	415
CCTL-14 low								
γH2AX	4.45 ± 0.20	554	12.83 ± 0.31	544	20.99 ± 0.34	634	30.37 ± 0.57	378
53BP1	2.73 ± 0.14	629	10.28 ± 0.36	508	16.77 ± 0.27	681	19.13 ± 0.39	452
CCTL-14 high								
γH2AX	6.66 ± 0.29	510	19.30 ± 0.49	427	16.26 ± 0.28	642	19.38 ± 0.34	423
53BP1	4.75 ± 0.21	576	14.34 ± 0.52	402	21.49 ± 0.30	653	24.20 ± 0.42	425

The number of foci per cell and the number of analysed cells are shown for non-irradiated control cells and cells 0.5 h after irradiation with 1 Gy. Five samples were analysed: a fibroblast line (hDF), three hiPSC lines (CBIA-3, CBIA-5, and CBIA-7) and one hESC line (CCTL-14) at high or low passage number. The values are shown for the separated cell cycle phases G1 (EdU-negative) or S/G2 (EdU-positive)

*EdU* 5-ethynyl-2′-deoxyuridine, *hDF* human dermal fibroblast

### hiPSCs lose their DNA repair capacity after prolonged maintenance in vitro

The above experiments demonstrated that, under normal culture conditions, hDFs, hiPSCs, and hESCs all have characteristic numbers of γH2AX and 53BP1 foci. However, based on these measurements we cannot resolve whether this is due to differences in the level of “spontaneous” DNA damage, DNA repair capability (recognition of DNA lesions), or both. It is understood that the amount of DSBs in cultured cells caused by γ-irradiation is about the same for the same dose of irradiation, regardless of the type of cell. With this holding true, the numbers of γH2AX and 53BP1 foci detected in cells irradiated by the same dose of γ-rays should then reflect the capability of the DNA repair machinery to recognize DSBs rather than the level of DNA damage. In the following series of experiments, we built on this presumption to study the DNA repair efficiency of human pluripotent stem cells. We irradiated the respective cells (hDFs, hiPSCs, and hESCs) with the same dose of γ-rays (1 Gy) and then determined the number of γH2AX and 53BP1 foci at three different time points after irradiation (0.5, 2, and 6 h). It has previously been shown that the levels of γH2AX and 53BP1 loaded onto chromatin usually reach a maximum at approximately 15–30 min after ionizing irradiation [32–34]. Based on this data, we used 30 min as the starting point. Two additional time points (2 and 6 h) then provided information on how DNA repair is sustained.

Figure 3c and d show the numbers of γH2AX and 53BP1 foci 30 min after γ-irradiation in EdU-negative cells. As expected for normal cells, hDFs exhibited a dramatic increase to 19.9 and 15.6, respectively. This represents an 18-fold (for γH2AX) and 10-fold (for 53BP1) increase over their numbers in non-irradiated controls, which indeed mirrors a massive initiation of DNA repair pathways (Fig. 4b). Surprisingly, although the numbers of both types of foci were higher in non-irradiated hiPSCs (irrespective of their passage number) than in hDFs (see the previous section), this was not the case for irradiated hiPSCs. Specifically, at 30 min after irradiation, low-passage hiPSCs produced 21.9 to 28.6 γH2AX foci and 17.8 to 20.1 53BP1 foci, thus always exceeding the corresponding numbers observed in hDFs. In contrast, high-passage hiPSCs produced only 8.0 to 12.4 γH2AX foci and 8.0 to 16.9 53BP1 foci. In other words, in hiPSCs, their prolonged passaging dramatically diminished the numbers γH2AX and 53BP1 foci induced by γ-rays to levels below or similar to those observed in hDFs.

As described in the previous section, the numbers of “spontaneous” γH2AX and 53BP1 foci were, for all cell types and categories studied here, always higher in S/G2 (EdU-positive) then in G1 (EdU-negative) cells. In hiPSCs, the fold-increase ranged from three-times in high-passage CBIA-5 cells (53BP1) to 4.6-times in high-passage CBIA-7 cells (γH2AX), and the changes were consistently statistically significant (Fig. 4a). This overall trend was also retained in γ-irradiated cells (at 30 min after irradiation); however, the actual fold-increase (S/G2 versus G1) was much lower, in four cases showing either no changes or statistically insignificant changes (for both γH2AX and 53BP1 foci in high-passage CBIA-3 and CBIA-7 hiPSCs) (Fig. 4a). Specifically, for hiPSCs, the fold-increase ranged from none to 2.1-times (24.6/11.6) for γH2AX foci in high-passage CBIA-5 cells. The percent increase of foci (both γH2AX and 53BP1) after treatment with 1 Gy was higher in the cells in G1 phase than in those in S/G2 phase (Fig. 4b). Taken together, this set of data reveals that a high level of spontaneous DNA damage (replicative stress occurring in S/G2 phase) dramatically distorts the outcome of γ-irradiation as measured by the numbers of γH2AX and 53BP1 foci.

**Fig. 5:**
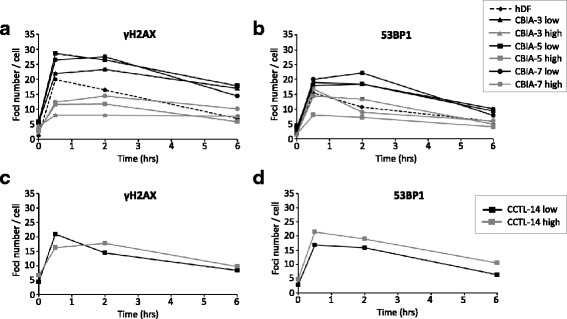
The time-course showing the recovery of human dermal fibroblasts (*hDFs*) and hiPSCs (**a**,**b**) or hESCs (**c**,**d**) after 1 Gy of γ-irradiation. **a**,**c** Number of DSBs recognized by γH2AX and **b**,**d** by p53-binding protein 1 (*53BP1*) in the untreated control (0 h) and at 0.5 h, 2 h, and 6 h after irradiation with 1 Gy. The results are shown in the EdU-negative population. The mean value of the DSBs was calculated for each of the three regions on the slide. The error bar indicates the SEM

As detailed above, we have found that irradiated high-passage hiPSCs load their DNA with much lower amounts of γH2AX and 53BP1 than irradiated hDFs and low-passage hiPSCs, suggesting that high-passage hiPSCs are somewhat less proficient at initiating DNA repair. To further examine this issue, we also determined the numbers of γH2AX and 53BP1 foci at 2 and 6 h after γ-irradiation and then analysed the shapes of the resulting time-course curves. The steepness of the resulting curves, which are shown in Fig. 5a and b, collectively confirm our initial notion. The curves representing hDFs and low-passage hiPSCs decline more steeply, indicating a faster decrease in DSBs, while the curves representing high-passage hiPSCs decline more gradually, indicating slower recovery from DSBs.

We also analysed hESCs in parallel to hiPSCs to determine whether the studied phenomena are associated with de-differentiation to pluripotency or with the pluripotency per se. Overall, the differences between irradiated low- and high-passage hESCs were much less pronounced than those in hiPSCs. This conclusion is substantiated by the numbers of: 1) γH2AX foci in low- and high-passage EdU-negative hESCs (21.0 versus 16.3); 2) 53BP1 foci in low- and high-passage EdU-negative hESCs (16.8 versus 21.5) (Fig. 3c and d); 3) γH2AX foci in low- and high-passage EdU-positive hESCs (30.4 versus 19.4); and 4) 53BP1 foci in low- and high-passage EdU-positive hESCs (19.1 versus 24.2) (Fig. 4a). It is of note that in high-passage hESCs (both EdU-negative and EdU-positive) the numbers of 53BP1 foci (but not of γH2AX foci) even increased compared to those typical for low-passage hESCs. Finally, the steepness of the time-course curves indicated that the decrease was more similar to hDFs than to hiPSCs (Fig. 5c and d).

**Fig. 6:**
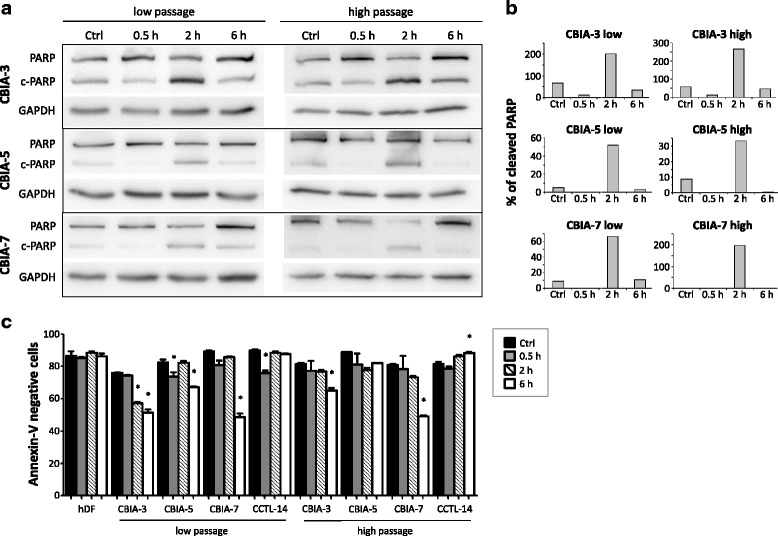
PARP expression and early apoptosis. Cells were exposed to 1 Gy radiation and analysed by Western blotting and flow cytometry after 0.5, 2, and 6 h. **a** Western blotting of PARP, cleaved PARP (*c-PARP*), and GAPDH. **b** Densitometric analysis shows the ratio of cleaved PARP to uncleaved PARP. **c** Annexin-V and 7-AAD were measured to assess the level of early apoptosis by flow cytometry. The Annexin-V- and 7-AAD-negative cell population is shown in the graph (± SEM). *P ˂ 0.05 versus control (*Ctrl*) within each group by one-way ANOVA and Tukey’s multiple comparison test). *hDF* human dermal fibroblast

To test possible differences in the sensitivity of particular cell types to apoptotic signals, we investigated the cleavage of PARP, an indirect marker of DNA damage, and early apoptosis using Annexin-V/7-AAD assay. A Western blotting analysis of PARP in hiPSC lines demonstrated that the highest cleavage occurred at 2 h after γ-irradiation (Fig. 6a and b). No difference was observed between low and high passages. The PARP cleavage was later accompanied by a decrease in cell viability at the 6-h time-point in all hiPSC lines with the exception of high-passage CBIA-5 cells. Interestingly, hDFs and hESCs did not display as much sensitivity to 1 Gy γ-irradiation as hiPSCs (Fig. 6c).

## Discussion

A DNA molecule is unstable and subject to internal and external harmful factors. Correct functioning of the DNA repair mechanisms is, therefore, essential for the maintenance of genomic integrity. In the field of hiPSC research, only cells with an intact genome can be used for clinical application. Unfortunately, the generation and expansion of hiPSCs in vitro causes genomic instability. In our research, we monitored the amount of DSBs, either spontaneous or irradiation-induced, in three lines of hiPSCs (CBIA-3, CBIA-5, and CBIA-7) at low or high passage numbers, as well as in original source cell fibroblasts (hDFs). One hESC line (CCTL-14) was also examined. Our goal was to shed light on the reaction of the cells to reprogramming and on the prolonged in vitro culturing of pluripotent cells. Here, we focused specifically on the kinetics of DSB generation and repair, cell cycle speed changes, triggering of apoptosis, and cell viability. Special attention was paid to the cell cycle phase of individual cells.

We selected two markers of DSBs—the phosphorylated histone variant γH2AX and its binding partner, the DNA repair mediator 53BP1. Fluorescence microscopy was chosen to detect these proteins because it offers two main advantages over other methods such as Western blotting. First, the expression of 53BP1 does not change; only its localization at DNA damage sites is affected. Second, analysis at the single-cell level assures a higher sensitivity and allows for the discrimination between cells at various cell cycle phases. We employed EdU staining, which discriminates between the G1 and S/G2 phases of the cell cycle. By incorporating a nucleoside analogue of thymidine (EdU) into the DNA during replication, only cells in the S or G2 stage are labelled positive [35]. The images were analysed using Acquiarium software. This software allows us to reliably determine the foci number together with the intensity of the EdU signal for each individual cell and to analyse the data from hundreds of cells per sample on a large scale. Our method for separating EdU-negative (G1 phase) cells from EdU-positive (S/G2 phase) cells is based on plotting the EdU intensity levels in histograms and using the Otsu method to find the threshold. Using this method, we revealed a longer cell cycle in somatic cells compared to pluripotent cells, which is in accordance with previously published data [36–39] and justifies the use of this method for cell cycle discrimination on the single-cell level. This approach also assures consistency among samples.

While counting the numbers of γH2AX and 53BP1 foci, it is of utmost importance to know exactly which phase of the cell cycle each individual cell is in at the moment. Our data show that cells in S/G2 phase contain more γH2AX and 53BP1 foci than cells in G1 phase and that this difference is more pronounced in non-irradiated controls. These foci emerge due to replication stress during S phase [12, 22, 23, 40]. The replication-related foci play a critical role in the comparison of DSB numbers, especially between different cell types. As long as the cells have a similar cell-cycle length (e.g. pluripotent cells at a similar passage number), the number of DSBs could be compared relatively well without using cell cycle discrimination. However, the following factors influence the cell cycle speed and should be considered: 1) pluripotent cells have been reported to have a shorter cell cycle than differentiated somatic cells [36–39]; 2) pluripotent cells at high passages may have an increased rate of proliferation [39, 41, 42]; and 3) irradiation induces cell cycle arrest through checkpoints [43–45]. We analysed the foci number separately for the EdU-negative and EdU-positive groups to eliminate the replication stress bias. Our data indicate a higher percent increase of foci upon γ-irradiation of cells in G1 phase, which are not burdened by replication-related foci. The cell-cycle dependency was confirmed for both γH2AX and 53BP1 markers. In general, fewer foci were detected for 53BP1 than for γH2AX, suggesting that 53BP1 is a less sensitive DSB marker with a lower capacity to recognize DSBs than γH2AX. It is known that the HR pathway plays a pivotal role during hiPSC generation [46], and 53BP1 promotes the NHEJ repair pathway while inhibiting the HR pathway [19–21]. In contrast, γH2AX influences both the NHEJ and HR pathways, and 53BP1 does not bind to all of the γH2AX foci [10, 11, 18].

Similar research was performed by Suchánková et al. [25], who measured the formation of γH2AX- and 53BP1-positive nuclear bodies in relation to cell cycle stages. They used genetically modified HeLa-Fucci cells, which are able to express RFP-Cdt1 in the G1 phase and GFP-geminin in the S/G2/M phases to discriminate among the cell cycle phases. They observed a higher number of γH2AX-positive repair foci in the G2 phase than in the G1 phase for both non-irradiated and γ-irradiated (5 Gy) HeLa cells. In contrast to our work, they did not observe such a difference for 53BP1. It is of note that different cell types as well as a different radiation dose (1 Gy) were used in our study compared to Suchankova et al., and it has been previously published that foci formation upon ionizing radiation may vary between cell types and is radiation-dose dependent [32, 34, 47, 48].

In our study, we worked with three unique hiPSC lines that were derived from two independent cell types (dermal fibroblasts and blood cells) and reprogrammed either by the Sendai virus or episomal vectors. This selection of samples enables us to generalize our conclusions for hiPSCs to a certain extent. To avoid bias caused by replication-related foci, we further analysed γH2AX and 53BP1 foci numbers only in the G1 (EdU-negative) subgroup. Our results indicate that spontaneously occurring DSBs are best recognized by both markers in hiPSCs at low passage, while fewer foci were observed in hiPSCs at high passage and in source fibroblasts. A low foci number in fibroblasts, therefore, is increased significantly after reprogramming into hiPSCs (either by Sendai virus or episomal vectors) and then decreases again after long-term in vitro passaging. Our results are consistent with recently reported data showing that H2AX plays a critical role in iPSC generation. Gonzáles et al. reported an increase in γH2AX during the cellular reprogramming of mouse embryonic fibroblasts independent of viral integration [46]. The HR pathway was confirmed to be essential for the error-free repair of DSBs in both genome-integrating and non-integrating reprogramming. The importance of H2AX at the early stage of reprogramming was also suggested by Wu et al. [49]. Our observations markedly resemble the results of copy number variation (CNV) measurements by Hussein et al. [50]. They concluded that most de novo-formed CNVs are present in early-passage hiPSCs, while fewer CNVs are found in late-passage hiPSCs and fibroblasts. There is a strong connection between CNVs and DSBs because deletions in subtelomeric regions have been shown to be highly sensitive to DSBs and are the major cause of chromosomal instability [51, 52]. Similar results were published by Laurent et al. [7] who reported a higher frequency of CNVs in pluripotent samples than in non-pluripotent samples and noticed that some of the deletions receded from the population over long-term passaging. Taken together, their data suggest that genomic instability is highest in low-passage hiPSCs, and CNVs vanish during multiple clonal-based passages because most of the mutations do not provide any advantage. However, certain growth-advantageous mutations—for example, defects in genes controlling the cell cycle—may be fixed in the population [5].

The abovementioned findings imply that more DSBs at low passages are detected as a consequence of reprogramming stress and disappear as the hiPSCs are adapted to in vitro conditions and clonally selected. However, the irradiation experiments revealed that the high-passage hiPSCs cannot recognize DSBs as effectively as hDF source cells, particularly by γH2AX. The lack of ability to recognize the irradiation-induced DSBs was also obvious in all three high-passage hiPSCs lines in the time-course study. These data suggest that hiPSCs lose their repair capacity over multiple passages in vitro. Similar results were published by Zhang et al. on one mouse iPSC line [29]. They confirmed the compromised DNA damage repair capacity of iPSCs compared with the respective source cells after γ-irradiation treatment but did not focus on the length of the in vitro culturing of iPSCs. For potential clinical applications, the length of in vitro culturing time should be reduced to as short as possible. However, a certain amount of time in vitro is unavoidable because of the reprogramming process itself, cell expansion, and clearance of the remaining reprogramming factors (viral particles or vectors).

Of note, low- and high-passage hiPSCs displayed similar apoptotic responses upon γ-irradiation. PARP cleavage peaked 2 h after irradiation, which led to an increase in early apoptosis after an additional 4 h in most of the hiPSC lines. These data suggest that, despite differences in DSB recognition, both low- and high-passage hiPSCs exert DNA protection mechanisms that trigger apoptosis in reaction to γ-irradiation. Increased apoptosis was not observed in somatic hDFs or in the hESC line CCTL-14, suggesting their lower sensitivity to DNA damage.

## Conclusions

This study addresses the question of hiPSC capability to repair their DNA using three independent lines of hiPSCs. It shows for the first time that: 1) reprogramming to pluripotency increases the number of DNA double-strand breaks (DSBs) as recognized by the γH2AX and 53BP1 proteins; 2) these DSBs are not due to replicative stress to DNA; and 3) their numbers become reduced during prolonged propagation after reprogramming. It also shows that prolonged passaging of hiPSCs is associated with a decrease in their DNA repair capacity and that this is not the case for the hESC line CCTL-14. From a technical point of view, this study documents that solid accuracy in analysing numbers of DSBs requires discrimination between the cells in G1 and S/G2 phases of their cell cycle. Collectively, hiPSCs at low passage contain more DSBs than hiPSCs at high passage, but they can repair them more efficiently.

## Additional files

Additional file 1: Figure S1. Immunocytochemistry of pluripotency markers. The pluripotency markers Oct-3/4, Sox2, Nanog, and SSEA4 are highly expressed in all three hiPSC lines used in this study (CBIA-3, CBIA-5, and CBIA-7). An anti-mouse secondary antibody conjugated with Alexa Fluor® 488 was used to detect Oct-3/4, Sox2, and SSEA4. An anti-rabbit secondary antibody conjugated with Alexa Fluor® 488 was used to detect Nanog. *Scale bar* = 100 μm. (PPTX 384 kb)

Additional file 2: Figure S2. Histological staining of a teratoma. Cell types representative of the three germ layers were detected by histological analysis in the CBIA-7 hiPSC cell line at passage number 26. (A) Glandular structures with secretory cells (endoderm); (B) mesenchymal cells (mesoderm); (C) cells with melanin (ectoderm); (D) glomerulus-like cells (mesoderm). (PPTX 32103 kb)

Additional file 3: Figure S3. Histograms of EdU signal intensity. The distribution of the EdU-negative and EdU-positive populations in hDF source cells, hiPSCs (CBIA-3, CBIA-5, and CBIA-7) and hESCs (CCTL-14) at low or high passage is shown. The samples were fixed 0.5 h, 2 h, and 6 h after 1 Gy of γ-irradiation. The thresholds for the EdU-negative population were calculated as described in the Methods section and are marked with a dotted line. (PPTX 251 kb)

## Abbreviations

53BP1: p53-Binding protein 1
7-AAD: 7-Amino-actinomycin D
ANOVA: One-way analysis of variance
CNV: Copy number variation
DSB: Double-strand break
EdU: 5-Ethynyl-2′-deoxyuridine
FITC: Fluorescein isothiocyanate
hDF: Human dermal fibroblast
hESC: Human embryonic stem cell
hiPSC: Human induced pluripotent stem cell
HR: Homologous recombination
IR: Ionizing radiation
MEF: Mouse embryonic fibroblast
NHEJ: Non-homologous end-joining
p: Passage
PSC: Pluripotent stem cell
