# Supplementary figures and images for: DNA double-strand breaks in human induced pluripotent stem cell reprogramming and long-term in vitro culturing
Source: Stem Cell Res Ther. 2017 Mar 21;8:73. doi: 10.1186/s13287-017-0522-5 (PMC5361733; doi:10.1186/s13287-017-0522-5)

## Slide 1
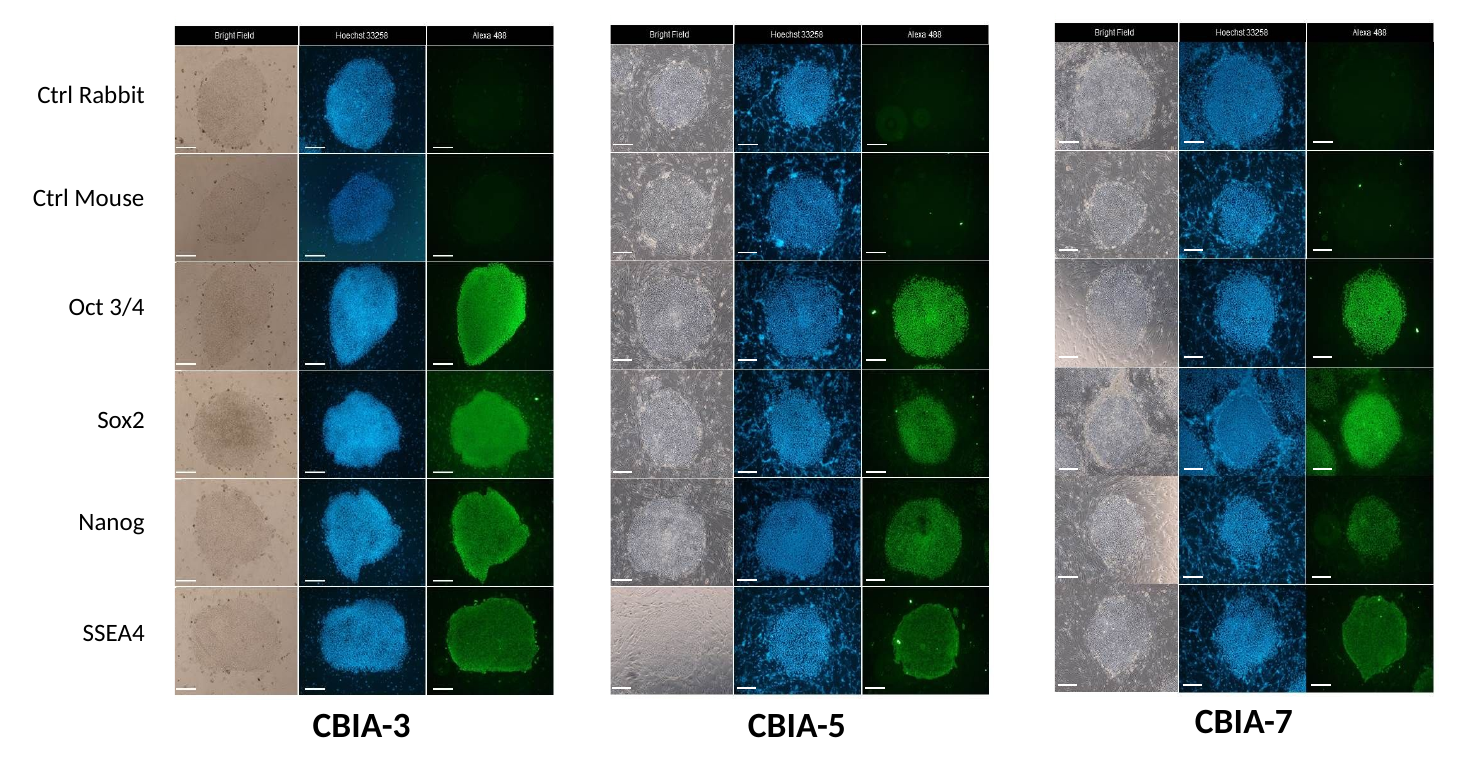

Ctrl Rabbit
Ctrl Mouse
Oct 3/4
Sox2
Nanog
SSEA4
CBIA-7
CBIA-3
CBIA-5

Supplement: Supplementary file 1 — Immunocytochemistry of pluripotency markers. The pluripotency markers Oct-3/4, Sox2, Nanog, and SSEA4 are highly expressed in all three hiPSC lines used in this study (CBIA-3, CBIA-5, and CBIA-7). An anti-mouse secondary antibody conjugated with Alexa Fluor® 488 was used to detect Oct-3/4, Sox2, and SSEA4. An anti-rabbit secondary antibody conjugated with Alexa Fluor® 488 was used to detect Nanog. Scale bar = 100 μm. (PPTX 384 kb) [file 13287_2017_522_MOESM1_ESM.pptx]

## Slide 1
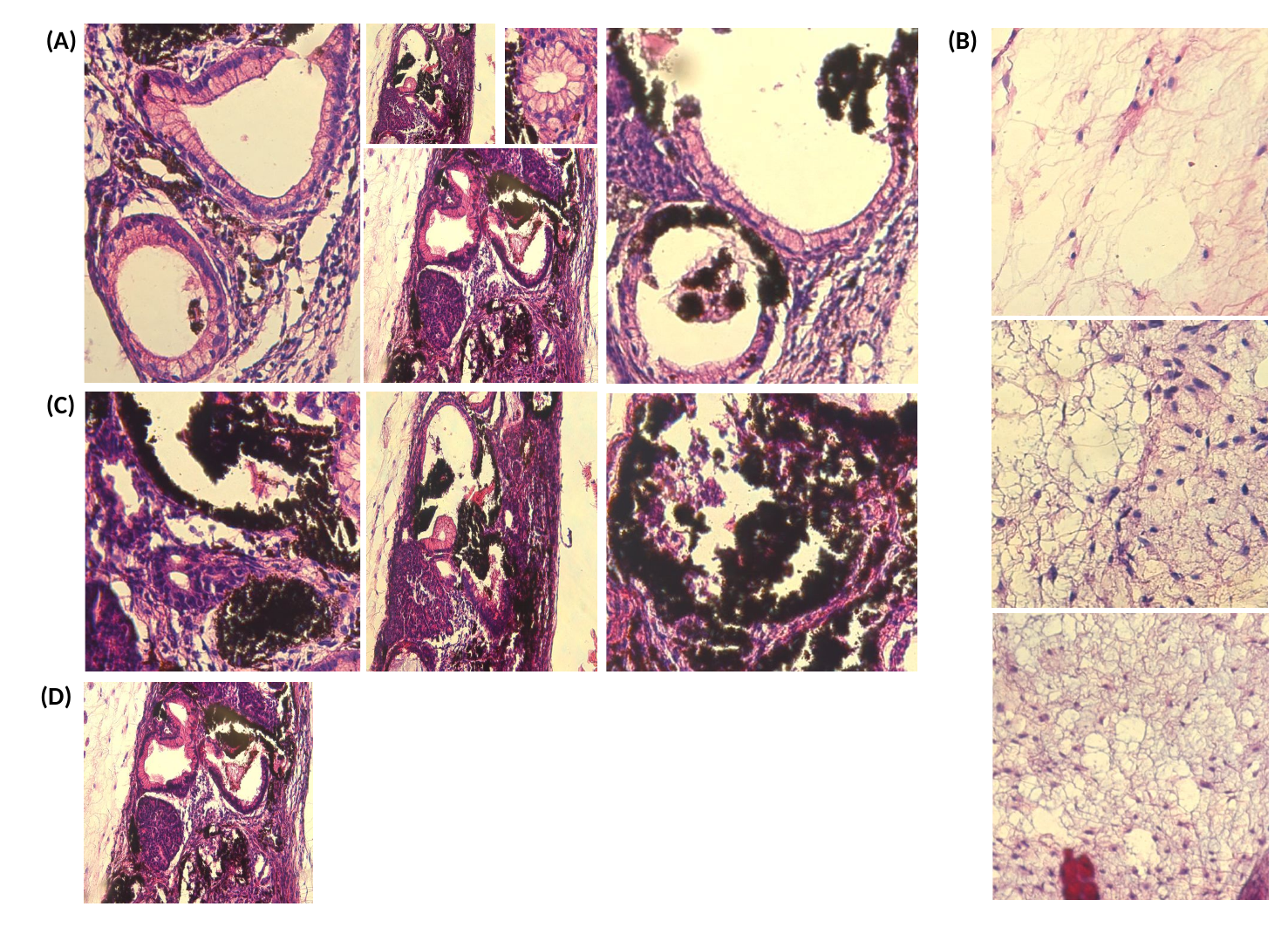

(A)
(B)
(C)
(D)

Supplement: Supplementary file 2 — Histological staining of a teratoma. Cell types representative of the three germ layers were detected by histological analysis in the CBIA-7 hiPSC cell line at passage number 26. (A) Glandular structures with secretory cells (endoderm); (B) mesenchymal cells (mesoderm); (C) cells with melanin (ectoderm); (D) glomerulus-like cells (mesoderm). (PPTX 32103 kb) [file 13287_2017_522_MOESM2_ESM.pptx]
