# Supplementary material for: DNA double-strand breaks in human induced pluripotent stem cell reprogramming and long-term in vitro culturing
Source: Stem Cell Res Ther. 2017 Mar 21;8:73. doi: 10.1186/s13287-017-0522-5 (PMC5361733; doi:10.1186/s13287-017-0522-5)
Supplement: Supplementary file 3 — Histograms of EdU signal intensity. The distribution of the EdU-negative and EdU-positive populations in hDF source cells, hiPSCs (CBIA-3, CBIA-5, and CBIA-7) and hESCs (CCTL-14) at low or high passage is shown. The samples were fixed 0.5 h, 2 h, and 6 h after 1 Gy of γ-irradiation. The thresholds for the EdU-negative population were calculated as described in the Methods section and are marked with a dotted line. (PPTX 251 kb) [file 13287_2017_522_MOESM3_ESM.pptx]

## Slide 1
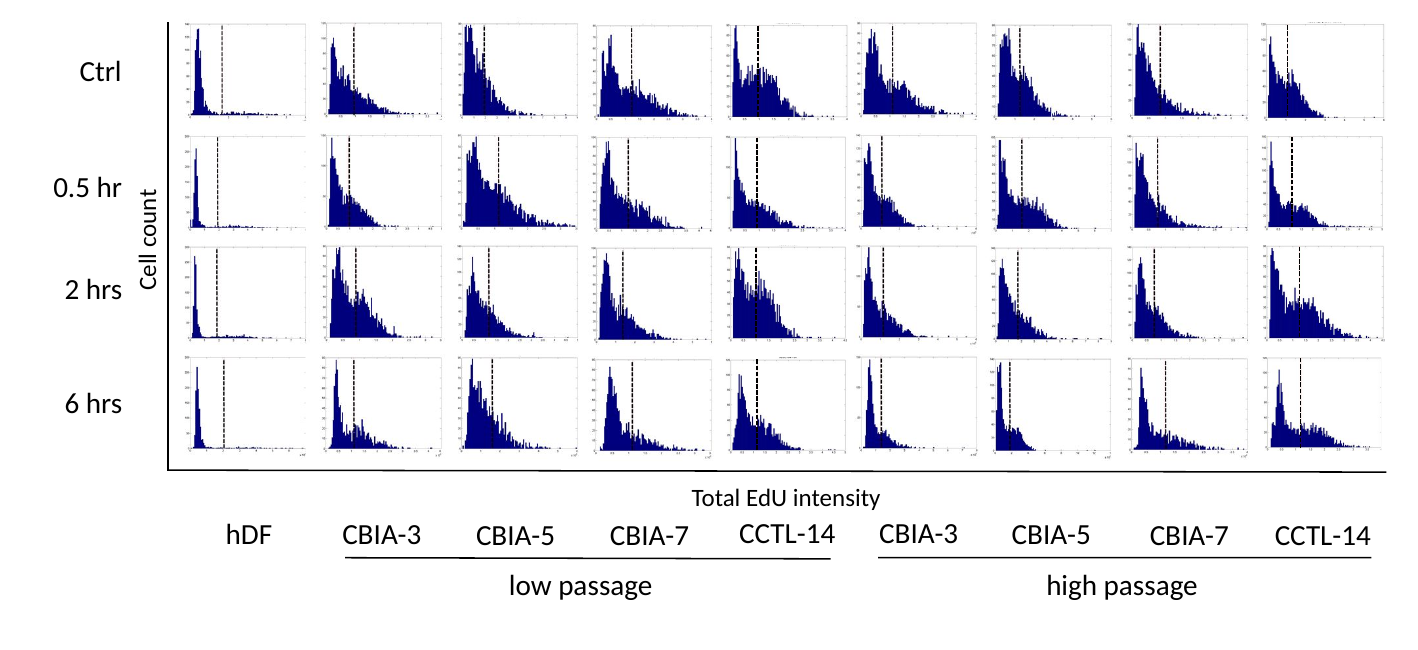

Ctrl
0.5 hr
Cell count
2 hrs
6 hrs
Total EdU intensity
CCTL-14
CBIA-3
hDF
CBIA-3
CBIA-5
CBIA-7
CCTL-14
CBIA-5
CBIA-7
low passage
high passage
